# Supplementary material for: Molecular insights into substrate translocation in an elevator-type metal transporter
Source: Nat Commun. 2024 Nov 8;15:9665. doi: 10.1038/s41467-024-54048-w (PMC11549095; doi:10.1038/s41467-024-54048-w)
Supplement: Supplementary file 2 — Description of Additional Supplementary Files [file 41467_2024_54048_MOESM2_ESM.pdf]

Description of Additional Supplementary Files

File Name: Supplementary Movie 1
Description: Illustration of the elevator-type transport mechanism of BbZIP.

File Name: Supplementary Data 1
Description: Includes oligos used in this work for mutagenesis.
