# Supplementary material for: Molecular insights into substrate translocation in an elevator-type metal transporter
Source: Nat Commun. 2024 Nov 8;15:9665. doi: 10.1038/s41467-024-54048-w (PMC11549095; doi:10.1038/s41467-024-54048-w)

## Slide 1
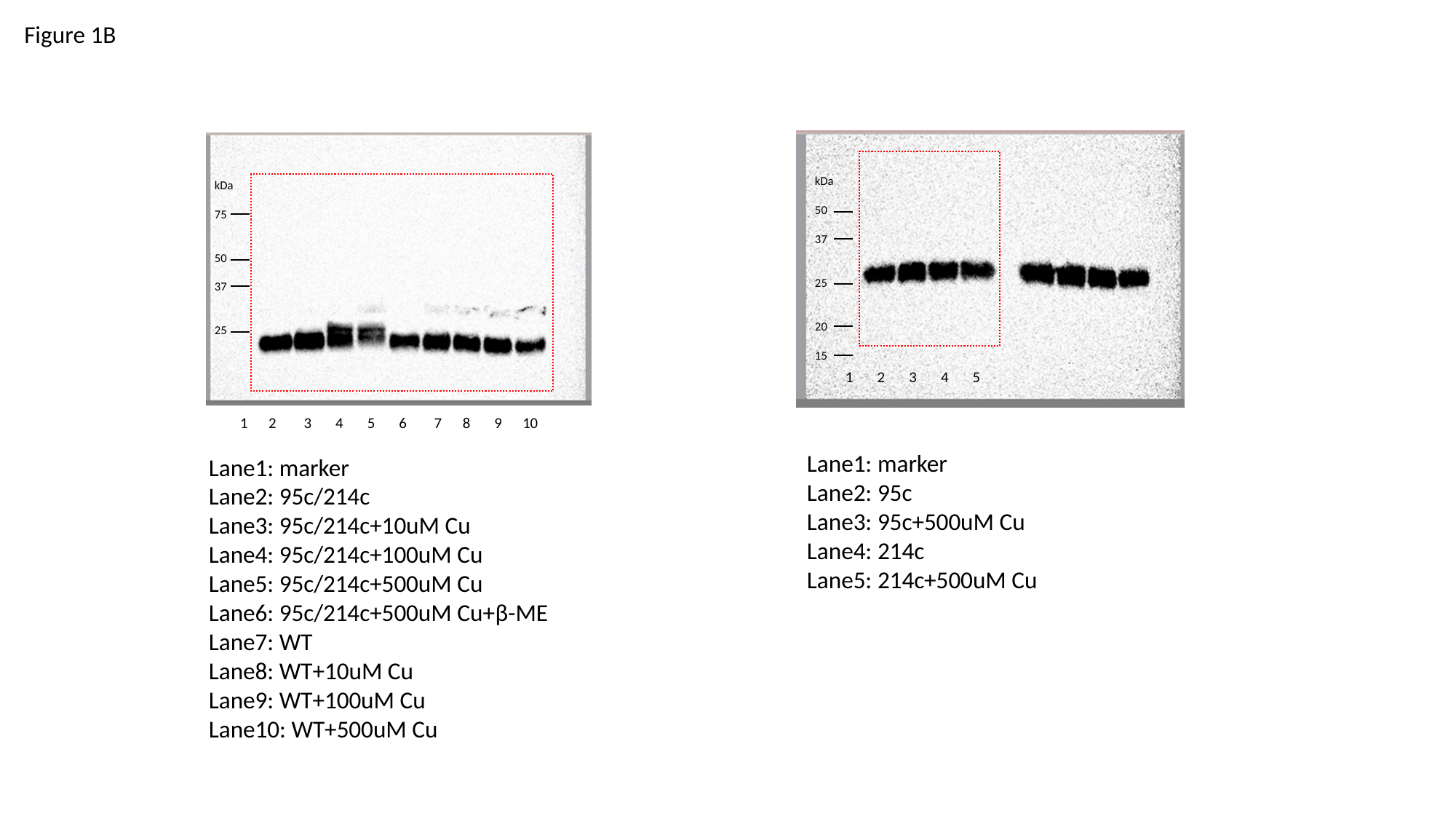

Figure 1B
kDa
75
50
37
25
kDa
50
37
25
20
15
1 2 3 4 5
1 2 3 4 5 6 7 8 9 10
Lane1: marker
Lane2: 95c
Lane3: 95c+500uM Cu
Lane4: 214c
Lane5: 214c+500uM Cu
Lane1: marker
Lane2: 95c/214c
Lane3: 95c/214c+10uM Cu
Lane4: 95c/214c+100uM Cu
Lane5: 95c/214c+500uM Cu
Lane6: 95c/214c+500uM Cu+β-ME
Lane7: WT
Lane8: WT+10uM Cu
Lane9: WT+100uM Cu
Lane10: WT+500uM Cu

## Slide 2
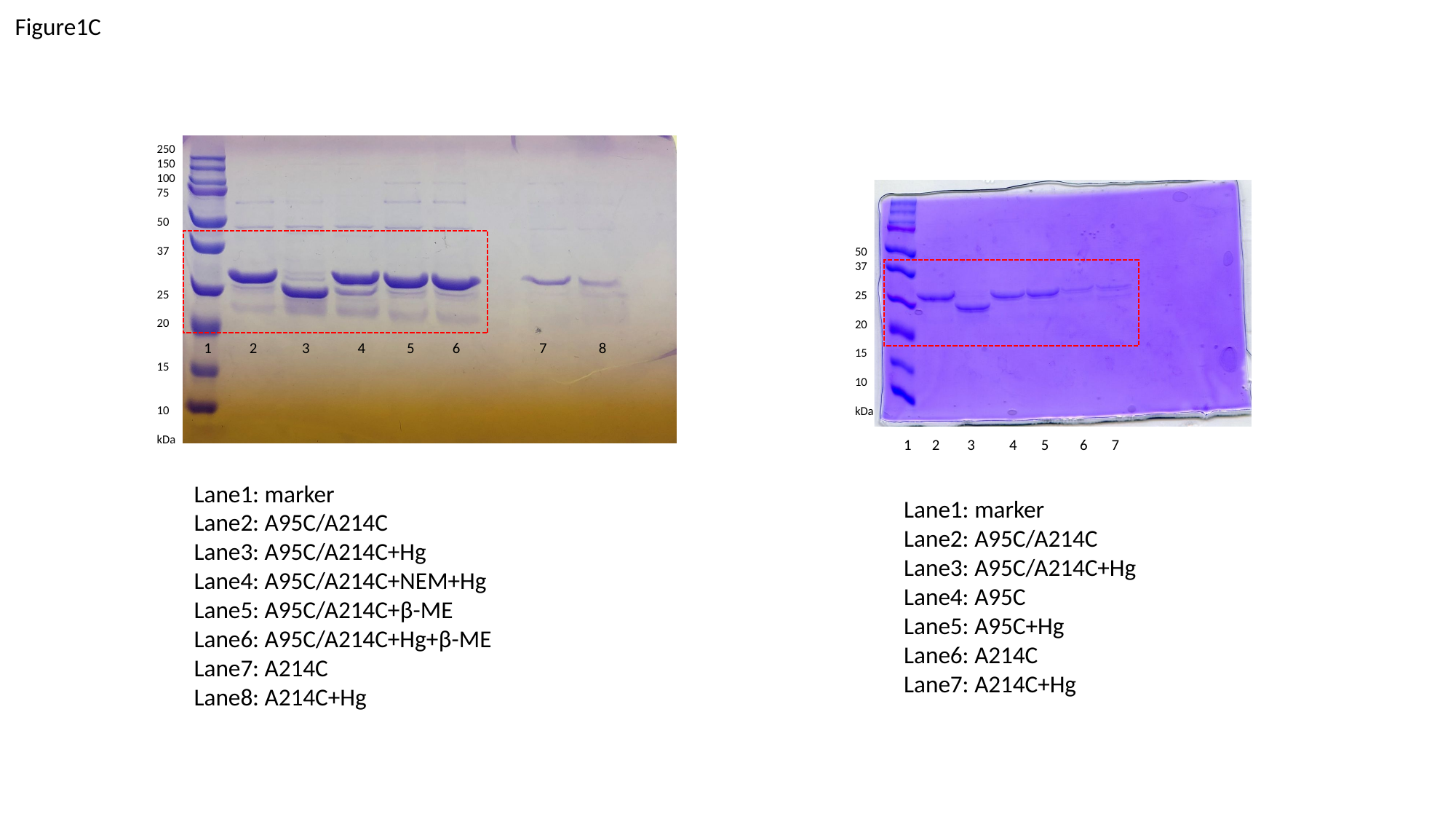

Figure1C
250
150
100
75
50
37
25
20
15
10
kDa
50
37
25
20
15
10
kDa
1 2 3 4 5 6 7 8
1 2 3 4 5 6 7
Lane1: marker
Lane2: A95C/A214C
Lane3: A95C/A214C+Hg
Lane4: A95C/A214C+NEM+Hg
Lane5: A95C/A214C+β-ME
Lane6: A95C/A214C+Hg+β-ME
Lane7: A214C
Lane8: A214C+Hg
Lane1: marker
Lane2: A95C/A214C
Lane3: A95C/A214C+Hg
Lane4: A95C
Lane5: A95C+Hg
Lane6: A214C
Lane7: A214C+Hg

## Slide 3
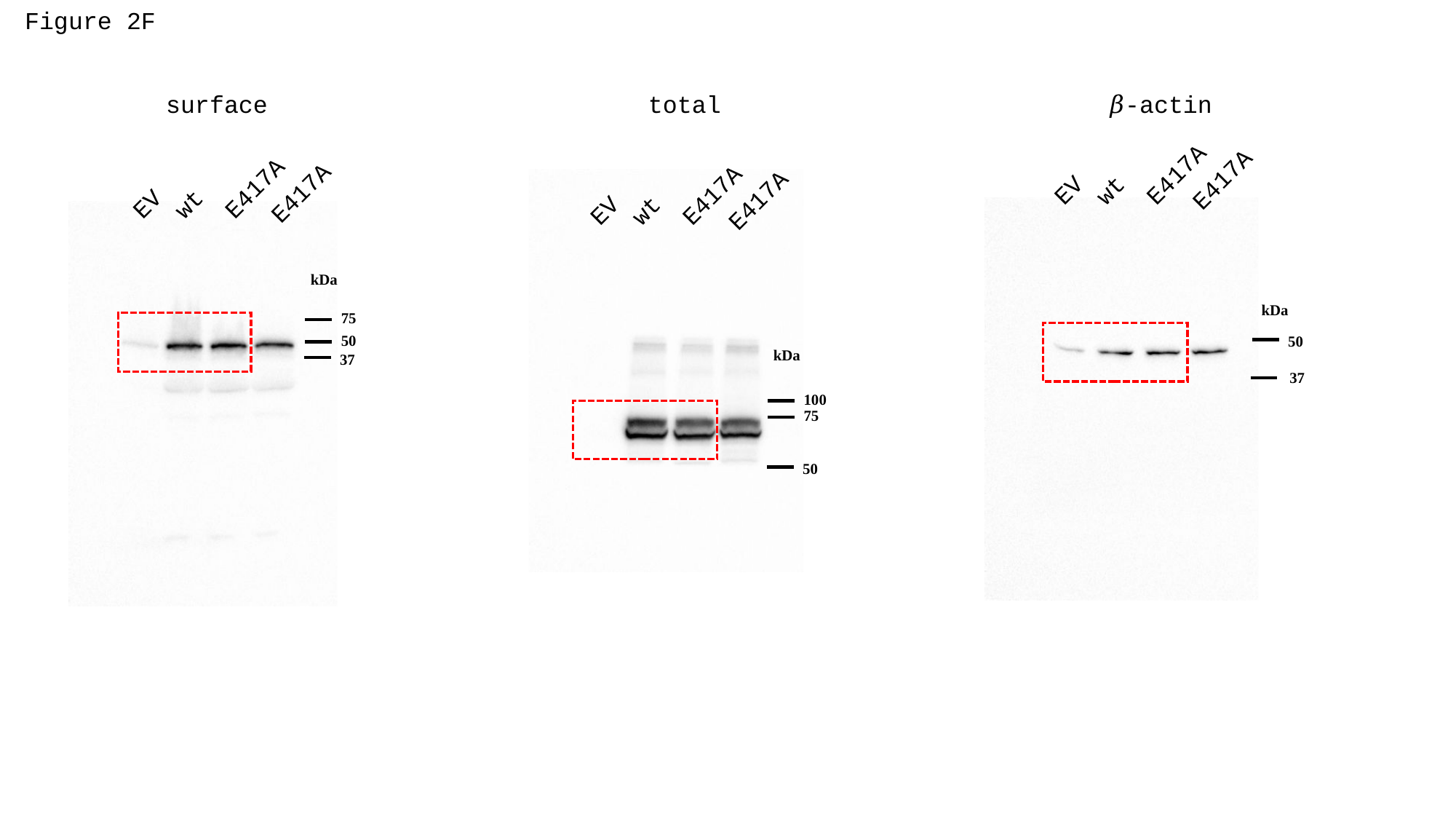

Figure 2F
surface
total
𝛽-actin
E417A
E417A
EV
wt
E417A
E417A
EV
wt
E417A
E417A
EV
wt
kDa
kDa
75
50
50
kDa
50
100
75
37
37

## Slide 4
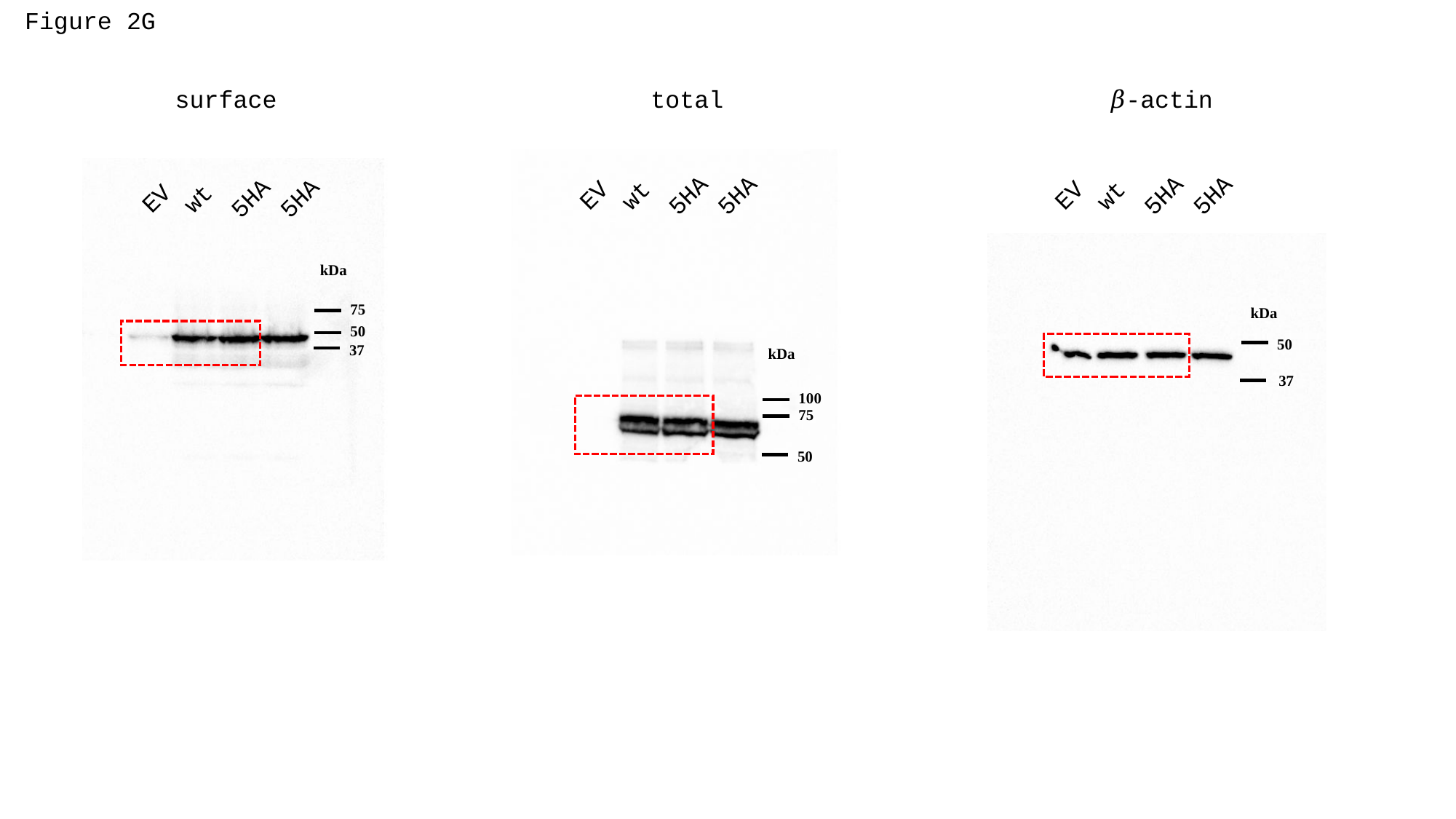

Figure 2G
surface
total
𝛽-actin
5HA
5HA
5HA
EV
wt
5HA
EV
wt
5HA
5HA
EV
wt
kDa
75
kDa
50
50
37
kDa
50
100
75
37

## Slide 5
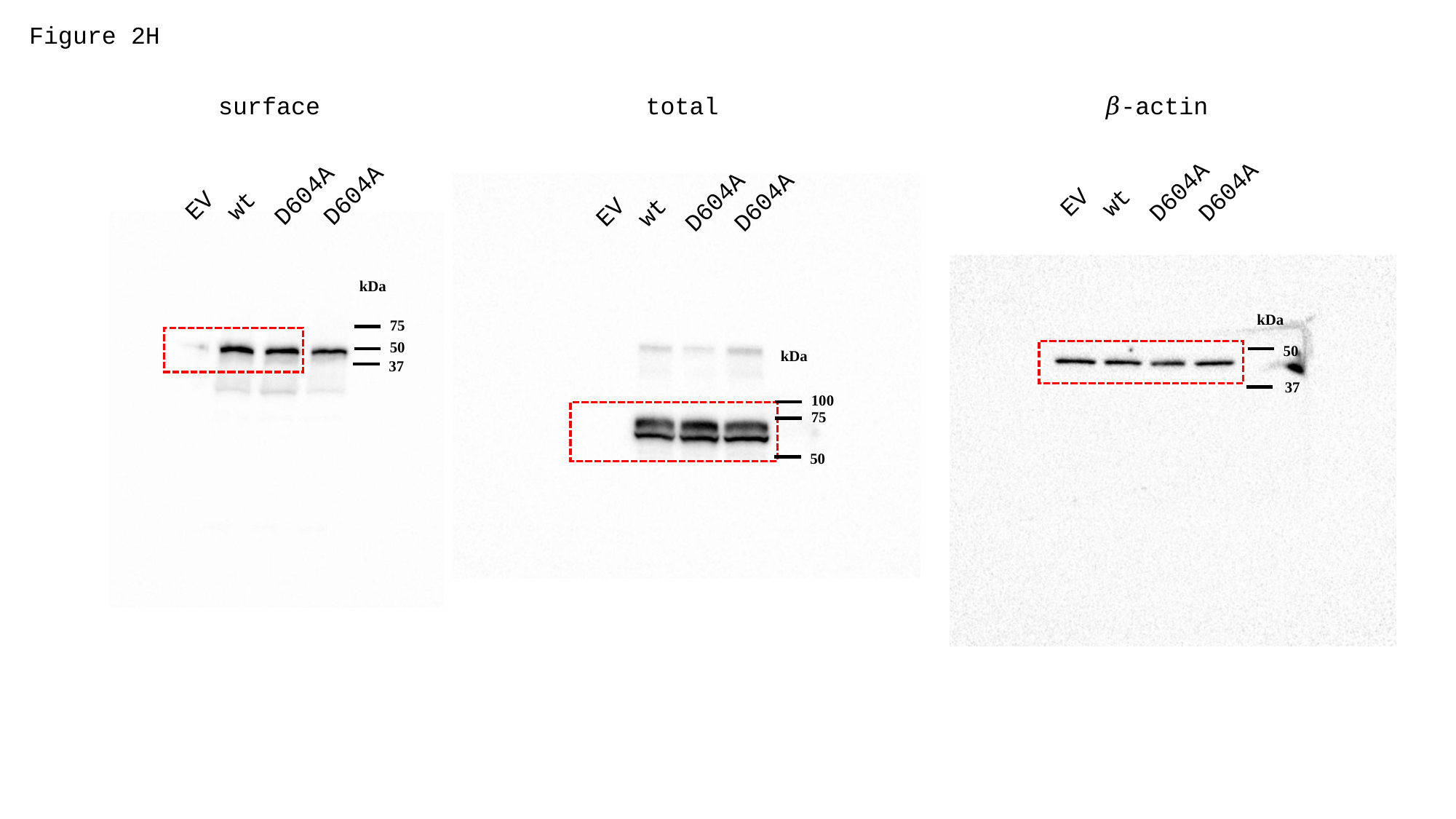

Figure 2H
𝛽-actin
surface
total
D604A
D604A
EV
wt
D604A
D604A
EV
wt
D604A
D604A
EV
wt
kDa
kDa
75
50
50
kDa
50
100
75
37
37

## Slide 6
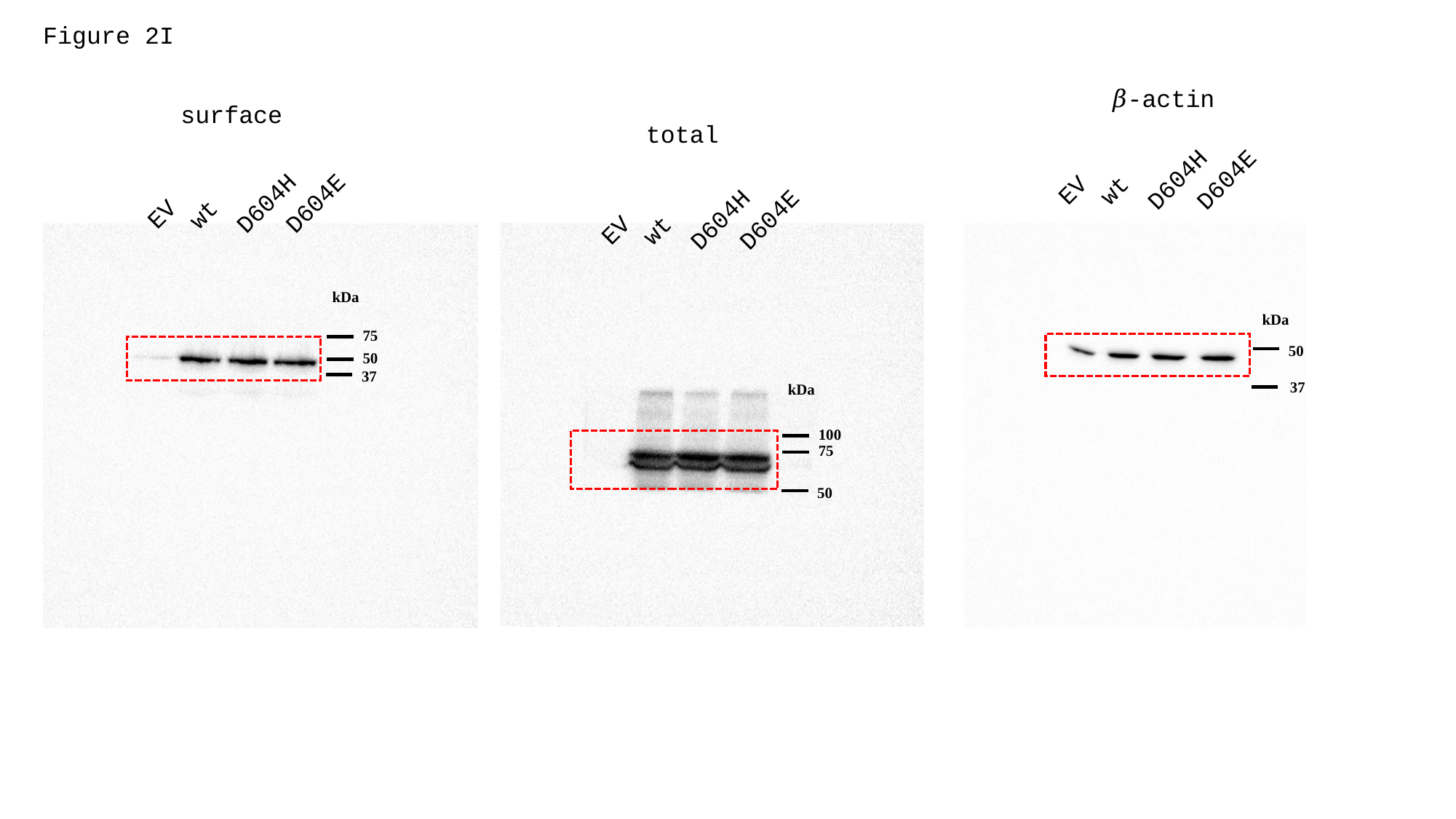

Figure 2I
𝛽-actin
surface
total
D604H
D604E
EV
wt
D604H
D604E
EV
wt
D604H
D604E
EV
wt
kDa
kDa
75
50
50
37
37
kDa
50
100
75

## Slide 7
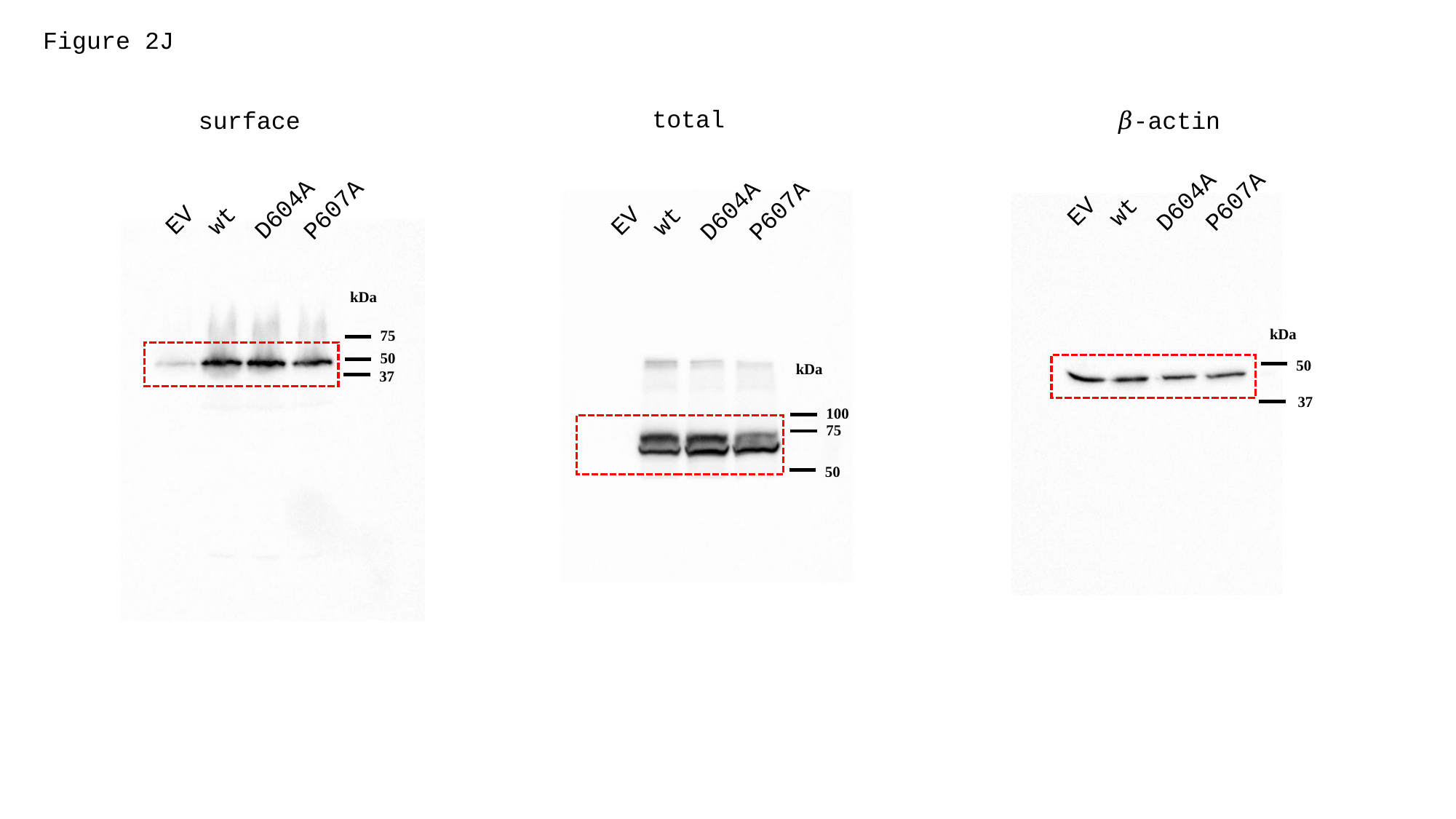

Figure 2J
total
surface
𝛽-actin
D604A
P607A
EV
wt
D604A
P607A
EV
wt
D604A
P607A
EV
wt
kDa
kDa
75
50
50
kDa
50
100
75
37
37

## Slide 8
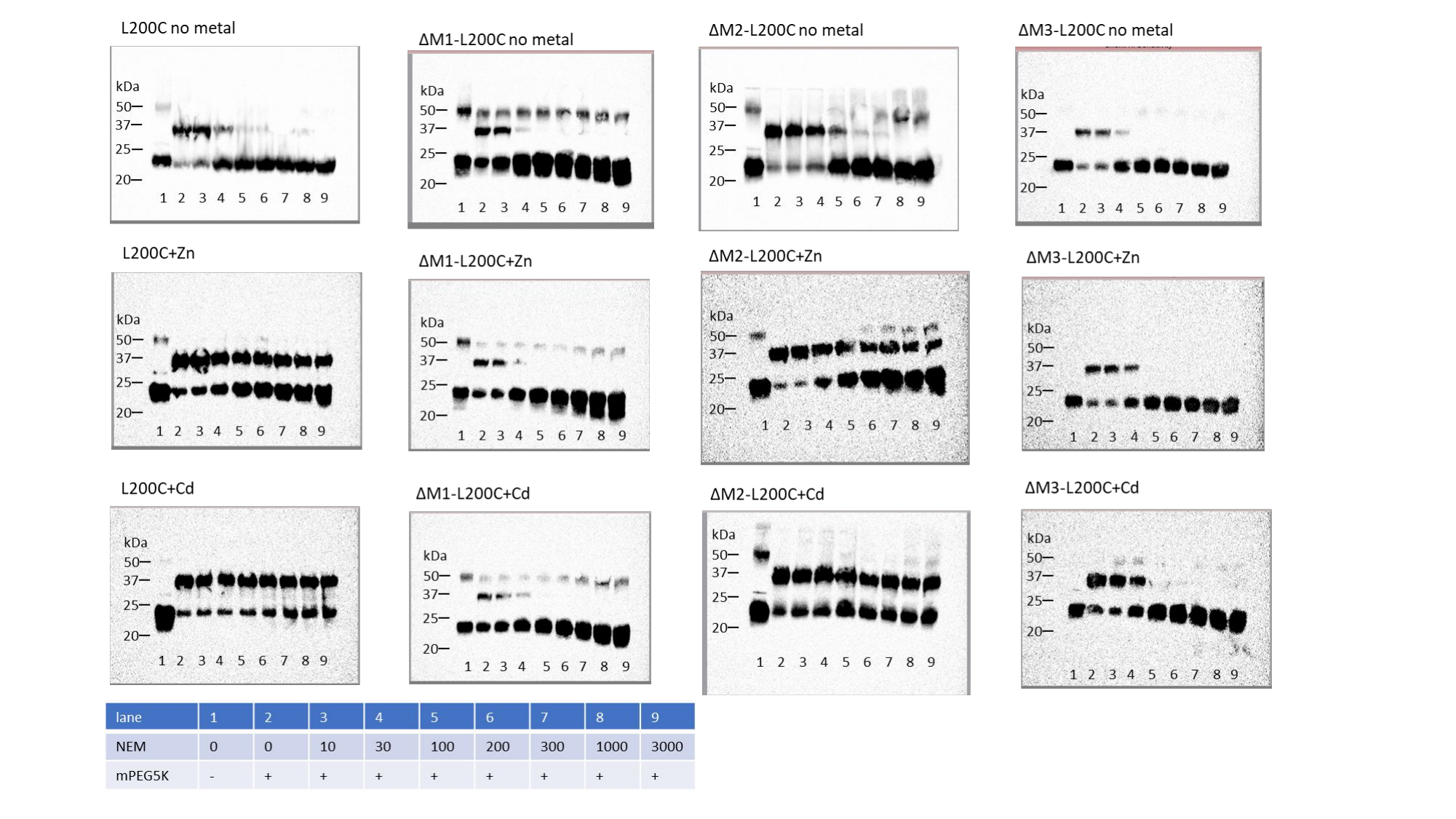

## Slide 9
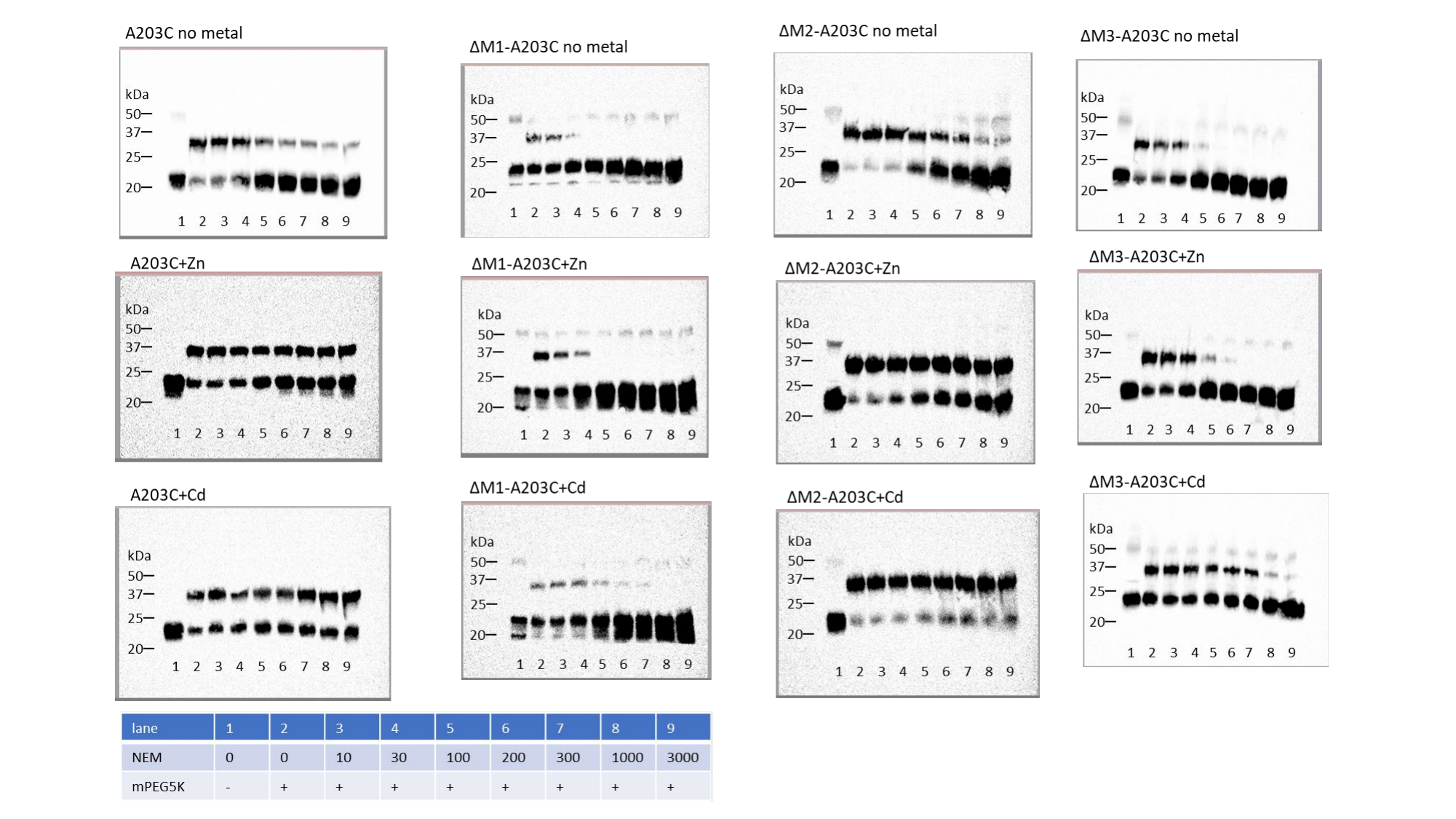

Supplement: Supplementary file 8 — Source Data 2 [file 41467_2024_54048_MOESM8_ESM.pptx]
